# Cesium lead bromide perovskite nanocrystals synthesized *via* supersaturated recrystallization at room temperature: comparison of one-step and two-step processes†

**DOI:** 10.1039/d4na00423j

**Published:** 2024-06-18

**Authors:** Dula Adugna Idosa, Mulualem Abebe, Dhakshnamoorthy Mani, Jibin Keloth Paduvilan, Lishin Thottathi, Aparna Thankappan, Sabu Thomas, Jung Yong Kim

**Affiliations:** a Faculty of Materials Science and Engineering, Jimma Institute of Technology, Jimma University P. O. Box 378 Jimma Ethiopia; b Department of Physics, College of Natural and Computational Science, Mizan-Tepi University P. O. Box 260 Mizan Ethiopia; c School of Chemical Sciences, Mahatma Gandhi University Kottayam 686560 India; d Department of Physics and Mathematics, Università Cattolica del Sacro Cuore Via della Garzetta, 48 25133 Brescia BS Italy; e Department of Physics, Baselius College Kottayam 686001 India; f School of Energy Materials, Mahatma Gandhi University Kottayam 686560 India; g Department of Materials Science and Engineering, Adama Science and Technology University P. O. Box 1888 Adama Ethiopia jungyong.kim@astu.edu.et; h Center of Advanced Materials Science and Engineering, Adama Science and Technology University P. O. Box 1888 Adama Ethiopia

## Abstract

Over more than a decade, lead halide perovskites (LHPs) have been popular as a next-generation semiconductor for optoelectronics. Later, all-inorganic CsPbX_3_ (X = Cl, Br, and I) nanocrystals (NCs) were synthesized *via* supersaturated recrystallization (SR) at room temperature (RT). However, compared to the hot injection (HI) method, the formation mechanism of NCs *via* SR-RT has not been well studied. Hence, this study will contribute to elucidating SR-RT based on the LaMer model and Hansen solubility parameter. Herein, we also demonstrate the entropy-driven mixing between two dissimilar polar-nonpolar (DMF–toluene) solvents. Next, we find that, in a poor solvent (toluene ≫ DMF in volume), ∼60 nm sized CsPbBr_3_ NCs were synthesized in one step, whereas in a marginal solvent (toluene ≈ DMF), ∼3.5 nm sized NCs were synthesized in two steps, indicating the importance of solvent polarity, specifically the ‘solubility parameter’. In addition, in the presence of a CuBr_2_ additive, high-quality cubic NCs (with ∼3.8 nm and ∼21.4 nm edge sizes) were synthesized. Hence, through this study, we present a ‘solubility parameter-based nanocrystal-size control model’ for SR-RT processes.

## Abstract 2

Over more than a decade, lead halide perovskites (LHPs) have been popular as a next-generation semiconductor for optoelectronics.

## Graphical abstract

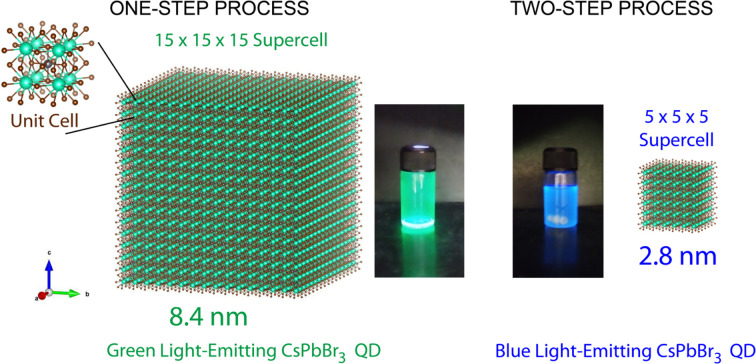

## 1. Introduction

All-inorganic cesium lead halide perovskite (LHP) (CsPbX_3_, X = Cl, Br, and I) nanocrystals (NCs), also called quantum dots (QDs) in the case of zero dimension (0D), have received increasing attention as emerging semiconductors in next-generation optoelectronic devices, including light emitting diodes (LEDs), solar cells, photodetectors,^1,2^ field-effect transistors (FETs),^3^ lasers, sensors,^4,5^ and quantum communication elements.^6^ Specifically, high color purity and high quantum efficiency make CsPbX_3_ NCs highly competitive for wide color gamut display applications.^7–10^ In addition to their outstanding luminescence performance, CsPbX_3_ NCs offer the advantage of tunable energy bands through chemical and morphology modulation.^11^

Green-emitting CsPbBr_3_ NCs were tuned to a blue emitter by adjusting the stoichiometry of halide (bromide and chloride) anions.^12,13^ However, LHP NCs with mixed halides are known to have drawbacks such as low defect tolerance of chlorine anions and phase instability upon exposure to light and/or voltage when applied as a blue light source in lighting and display technology.^13^ The other strategy for blue-emitting LHP NCs relies on the quantum confinement effect, a unique property of low-dimensional semiconductors. For example, strongly quantum-confined CsPbBr_3_ NCs, such as dots, nanowires, nanoplatelets, and nanocubes, have been demonstrated as blue emitters.^10,14–17^ However, the fast nucleation and growth rate of CsPbBr_3_ NCs, originating from their low particle formation energy and soft ionic lattice structure, makes it difficult to control the size and morphology of NCs in the highly quantum confined region.^18^

To address the precise control of the size and shape of LHP NCs, several processing methods such as ‘hot-injection (HI)’, ‘ligand-assisted reprecipitation (LARP)’, and ‘room-temperature (RT) supersaturated recrystallization (SR)’ have been employed.^10,19,20^ For example, Kovalenko and coworkers controlled the size of CsPbX_3_ NCs in the range of 4–15 nm edge lengths by varying the reaction temperature from 200 to 140 °C in 2015.^10^ Rogach *et al.* demonstrated the size-tuned bandgap of CH_3_NH_3_PbBr_3_ NCs by varying the precipitation temperature from 0 to 60 °C *via* LARP routes.^19^ Later, in 2016, Zeng and coworkers invented an RT-SR (a special case of LARP) method for the synthesis of CsPbX_3_ NCs within a few seconds at ambient conditions without any inert gas and local injection operation.^20^ Furthermore, ligand composition and LHP precursor concentration were varied to control the size of the NCs.^21,22^ Son *et al.* accurately controlled the CsPbX_3_ NC size with high ensemble uniformity utilizing thermodynamic equilibrium.^18^ Pradhan *et al.* reported the precise step-growth process of CsPbBr_3_ NCs *via* unit cell size (∼0.6 nm) increment.^16^ Zhang *et al.* demonstrated the size and shape control of LHP NCs using suitable amounts of water, contrary to the common preconception that LHPs may rapidly decompose when exposed to polar solvents such as water.^23^ Interestingly, Yang *et al.* achieved the controlled synthesis of ∼3 nm sized CsPbBr_3_ NCs using the cryogenic temperature synthetic strategy.^24^ Moreover, the quantum-confinement effect is further tunable by using copper, nickel, tin, cadmium, zinc, and aluminum ions as an additive (dopant) for CsPbX_3_ NCs.^25–28^

The nucleation and growth processes of LHP^29,30^ and cadmium chalcogenide (CdX, X = S, Se, and Te)^31,32^ NCs have been interpreted based on the classical LaMer model introduced in the 1950s.^33,34^ However, studies on the formation mechanism of colloidal NCs have been mostly focused on the popular HI^10,35^ and heat-up^31,32^ processes instead of the SR (LARP)^19,20,36^ method operating at RT. Hence, this work is dedicated to elucidating the NC formation mechanism for the green- and blue-emitting CsPbBr_3_ NCs synthesized *via* one-step and two-step SR processes at RT, respectively. For this purpose, we additionally employ, for the first time, the Hildebrand and Hansen solubility parameters^37^ determining the NC size (green or blue emitter) through the balance between the formation and dissolution of CsPbBr_3_ NCs in solvent medium – good (polar), marginal (partially polar) or poor (nonpolar). We find that the partially-polar marginal solvent medium is suitable for blue-emitting NCs whereas the nonpolar poor solvent quality is acceptable for green-emitting NCs. Furthermore, to understand the solvent–antisolvent (*e.g.*, the binary DMF–toluene system) miscibility, we employ the Flory–Huggins theory^38,39^ predicting the entropy-driven mixing between two dissimilar polar–nonpolar solvents, enabling the SR routes using solvent–antisolvent engineering for the synthesis of CsPbBr_3_ NCs at RT.

## 2. Materials and methods

### 2.1. Chemicals

Cesium bromide (CsBr, 99.9%, Sigma-Aldrich, Darmstadt, Germany), lead(ii) bromide (PbBr_2_, 99.0%, AR chemicals, Delhi, India), copper(ii) bromide (CuBr_2_, 99.9% Sigma-Aldrich, Darmstadt, Germany), oleic acid (OA, 98%, Sigma-Aldrich, Darmstadt, Germany), oleylamine (OAm, technical grade 70%, Sigma-Aldrich, Darmstadt, Germany), *N*,*N*-dimethylformamide (DMF, 99%, SRL, Mumbai, India), toluene (99%, Loba Chemie, Mumbai), and ethyl acetate (EA, 99%, SRL, Mumbai, India) were used as received without further purification.

### 2.2. Synthesis of CsPbBr_3_ NCs: one-step process

The precursor solution was prepared by dissolving PbBr_2_ (0.2 mmol), CsBr (0.2 mmol), OA (0.5 mL), and OAm (0.25 mL) in DMF (5 mL) under stirring for 2 hours at room temperature. Then, 1 mL of the solution was added to 10 mL toluene (*i.e.*, toluene ≫ DMF in volume) under vigorous stirring. Immediately, the formation of CsPbBr_3_ NCs was verified with a bright green-light emission in the solution under a 365 nm UV lamp.

### 2.3 Synthesis of CsPbBr_3_ NCs: two-step process

The precursor solution was prepared by dissolving PbBr_2_ (0.2 mmol), CsBr (0.2 mmol), OA (0.5 mL), and OAm (0.25 mL) in DMF (5 mL) under stirring for 2 hours at room temperature. Then, 5 mL of toluene was added to the 5 mL solution (toluene ≈ DMF in volume) under vigorous stirring to obtain the first stage of crystallization. Then, after aging for 2 min, 1 mL of the solution was added to 10 mL toluene (toluene ≫ DMF) to finalize the crystallization – the second stage in the two-step process. Immediately, the formation of CsPbBr_3_ NCs was verified with a bright blue-light emission in the solution under a 365 nm UV lamp. Note that all the synthesis reactions were carried out without any inert gas in the air.

### 2.4. Purification

For comparison purposes, the solutions of both NCs synthesized by one-step and two-step processes were centrifuged at 9000 rpm for 5 min, and the precipitates were collected and characterized. In this report, for the two-step synthesis, the precipitant is referred to as the unpurified CsPbBr_3_ NCs. To purify the NCs synthesized by two steps, the solution was first centrifuged at 3500 rpm to separate larger NCs and/or aggregation as precipitate and smaller ones as supernatant. The supernatant was collected and further centrifuged at 8500 rpm for 10 min to separate NCs from the solution, and the NCs are referred to as the purified CsPbBr_3_ NCs in this report. Finally, the supernatant was discarded, and the precipitate was dispersed in toluene for further characterization. In this study, CsPbBr_3_ NCs synthesized *via* the one-step process were used without further purification.

### 2.5. Characterization

The ultraviolet-visible (UV-Vis) absorption spectra of as-prepared NCs in toluene solution were obtained using the P9 UV-Vis spectrophotometer, China. The luminescence of NCs was recorded by the Cary Eclipse Fluorescence spectrophotometer, Malaysia. The crystal structure of the NCs was examined by X-ray diffraction (XRD) with Cu Kα radiation, *λ* = 1.5406 Å at 30 kV and 25 mA (Drawell XRD 7000, China). The morphology and microstructure as prepared NCs were analyzed by high-resolution transmission electron microscopy (HR-TEM) (JEOL JEM-2100; Peabody, MA, USA) with an operating voltage of 200 kV.

### 2.6. Computational details

In order to understand the electronic structures of CsPbBr_3_ perovskite, the density functional theory (DFT)-based first-principle calculations as in the Quantum ESPRESSO package were carried out.^40^ The generalized gradient approximation (GGA) functional of Perdew–Burke–Ernzerhof for solids (PBEsol) is used to describe the exchange–correlation potential.^41,42^ The interactions between the atomic core and the valence electrons were described by the ultrasoft pseudopotentials. The valence electronic configurations for Cs, Pb, and Br atoms are 5s^2^5p^6^6s^1^, 5d^10^6s^2^6p^2^ and 4s^2^4p^5^, respectively. A plane wave cut off 38 Ry and 7 × 7 × 7 *k*-point mesh was used in the calculation process. Accordingly, the electronic band structure and the projected density of state (PDOS) of CsPbBr_3_ are summarized and displayed in Fig. S1 in ESI.†

## 3. Results and discussion

**Fig. 1:**
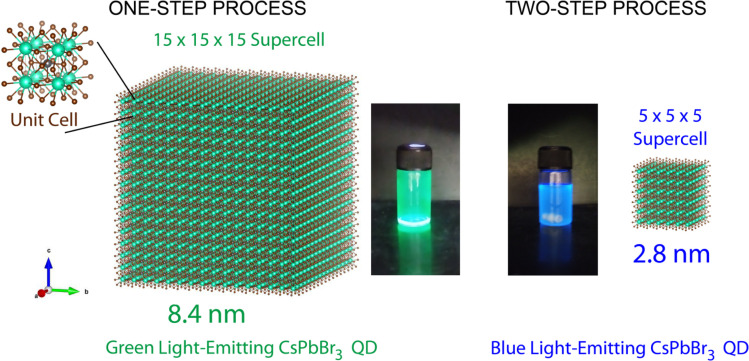
CsPbBr_3_ NCs with a cubic structure: green and blue-emitting NCs depending on the processing conditions: one-step *vs.* two-step. Unit-cell edge length is 0.587 nm.

Fig. 1 shows a schematic representation of the quantum confinement effect, *e.g.*, size-dependent light emission, of CsPbBr_3_ NCs. This phenomenon is observable when the electron and hole wave functions are reduced to be smaller than the excitonic Bohr radius, *e.g.*, ∼7 nm for CsPbBr_3_.^10,43–45^ Specifically, when the size of CsPbBr_3_ NCs is less than ∼4 nm, the pure blue emission could be expected.^46^ Hence, this study focuses on the processing-nanostructure-optical property relationship of these CsPbBr_3_ NCs with emphasis on the formation mechanism of green- and blue-emitting CsPbBr_3_ NCs *via* the SR method at RT.

**Fig. 2:**
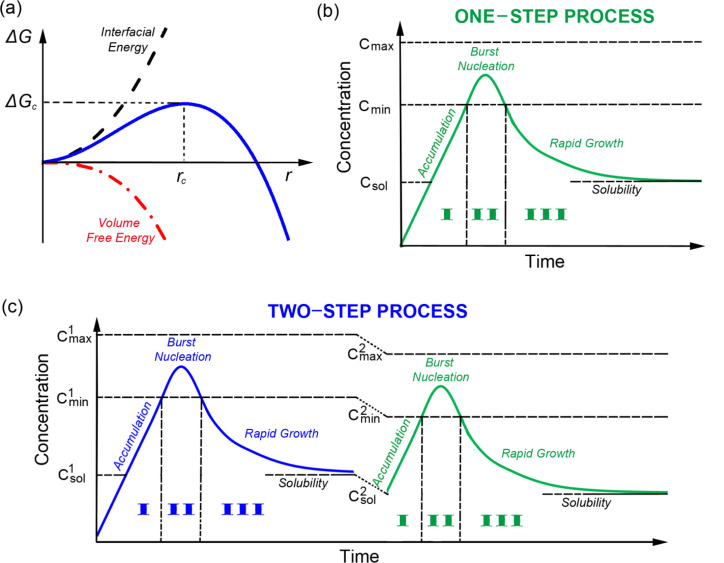
(a) Free energy change *vs.* particle size. LaMer diagram: (b) one-step process and (c) two-step process. The regions I, II and III correspond to (monomer) accumulation, burst nucleation and rapid growth, respectively.

Briefly, in the LaMer model (Fig. 2a),^33,34^ the total free energy (Δ*G*) for the formation of a spherical particle with radius *r* could be expressed as1
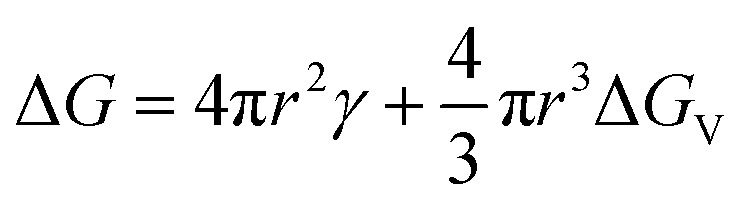
where *γ* is the solid/liquid interfacial energy per unit area and Δ*G*_V_ is the volume free energy. Here, Δ*G*_V_ can be expressed as −*k*_B_*T* ln *S*/*V*, where *k*_B_, *T*, and *V* are Boltzmann constant, temperature, and the volume of monomer (*e.g.*, Cs[PbBr_3_] aggregate or precursor ions/complexes in this study), respectively. Thus, the degree of supersaturation (*S*) is the driving force for the reduction of Δ*G*_V_. Under unstable equilibrium, ∂Δ*G*/∂*r* = 0, the critical radius is *r*_c_ = −2*γ*/Δ*G*_V_ = 2*γV*_m_/*RT* ln *S* and concomitantly, the total free energy Δ*G*(*r* = *r*_c_) is Δ*G*_c_ = 16π*γ*^3^/3Δ*G*_V_^2^ = 16π*γ*^3^*V*^2^/[3*k*_B_^2^*T*^2^(ln *S*)^2^]. Then, the nucleation rate (d*N*/d*t*) could be defined as follows,^31,32^2
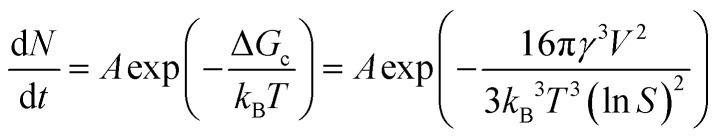
which describes that the burst nucleation is largely governed by the degree of supersaturation (*S*) and interfacial energy (*γ*) in the case of SR at RT (constant temperature). Here, *A* is a prefactor. Importantly, the factor *S* can be controlled by the amount of nonpolar antisolvent (typically, toluene) at SR-RT. According to eqn (2), when a large amount of antisolvent is added to the perovskite precursor solution, *S* will increase, resulting in a high rate of nucleation.

Fig. 2b and c show the LaMer diagrams for the one- and two-step syntheses, respectively, which are composed of three regions, ‘Cs[PbBr_3_] aggregate’ accumulation (I), burst nucleation (II) and rapid growth (III), followed by the Ostwald ripening process. In this study, the ‘one-step process’ indicates that the perovskite precursor in polar DMF (1 mL) was injected into 10 mL of the nonpolar antisolvent toluene at RT, resulting in the green-emitting NC synthesis *via* a high degree of supersaturation.

On the other hand, the ‘two-step process’ denotes the sequential mixing of the perovskite precursor solution with the antisolvent. The first stage is the mixing of 5 mL DMF (perovskite precursor) and 5 mL toluene, resulting in the blue-emitting NC synthesis *via* a low degree of supersaturation. However, for collecting these blue emitters, the second stage is usually required. For example, 1 mL of the first stage sample is mixed with 10 mL of antisolvent (Fig. 2c), resulting in a bimodal distribution of NCs, *i.e.*, the blue and green emitters. Note that in Fig. 2c, the solubility of the eventual solvent systems (marginal *vs.* poor) is different in the first and second stages, affording low and high degrees of supersaturation, respectively. Hence, two different sets of monomer concentrations (*C*_max_^1^, *C*_min_^1^, and *C*_sol_^1^ for the 1st stage; and *C*_max_^2^, *C*_min_^2^, and *C*_sol_^2^ for the 2nd stage) should be considered. Here, *C*_max_ is the maximum supersaturation concentration, *C*_min_ is the minimum supersaturation concentration (*i.e.*, the critical monomer concentration), and *C*_sol_ is the solubility limit for a given solvent system (hence, the solubility parameter is essential for the SR-RT process). The superscripts 1 and 2 in the concentration (*C*) symbol denote the first and second stages, respectively. Furthermore, *S* can be expressed by *C*/*C*_sol_ as long as *C* is larger than *C*_sol_.

**Fig. 3:**
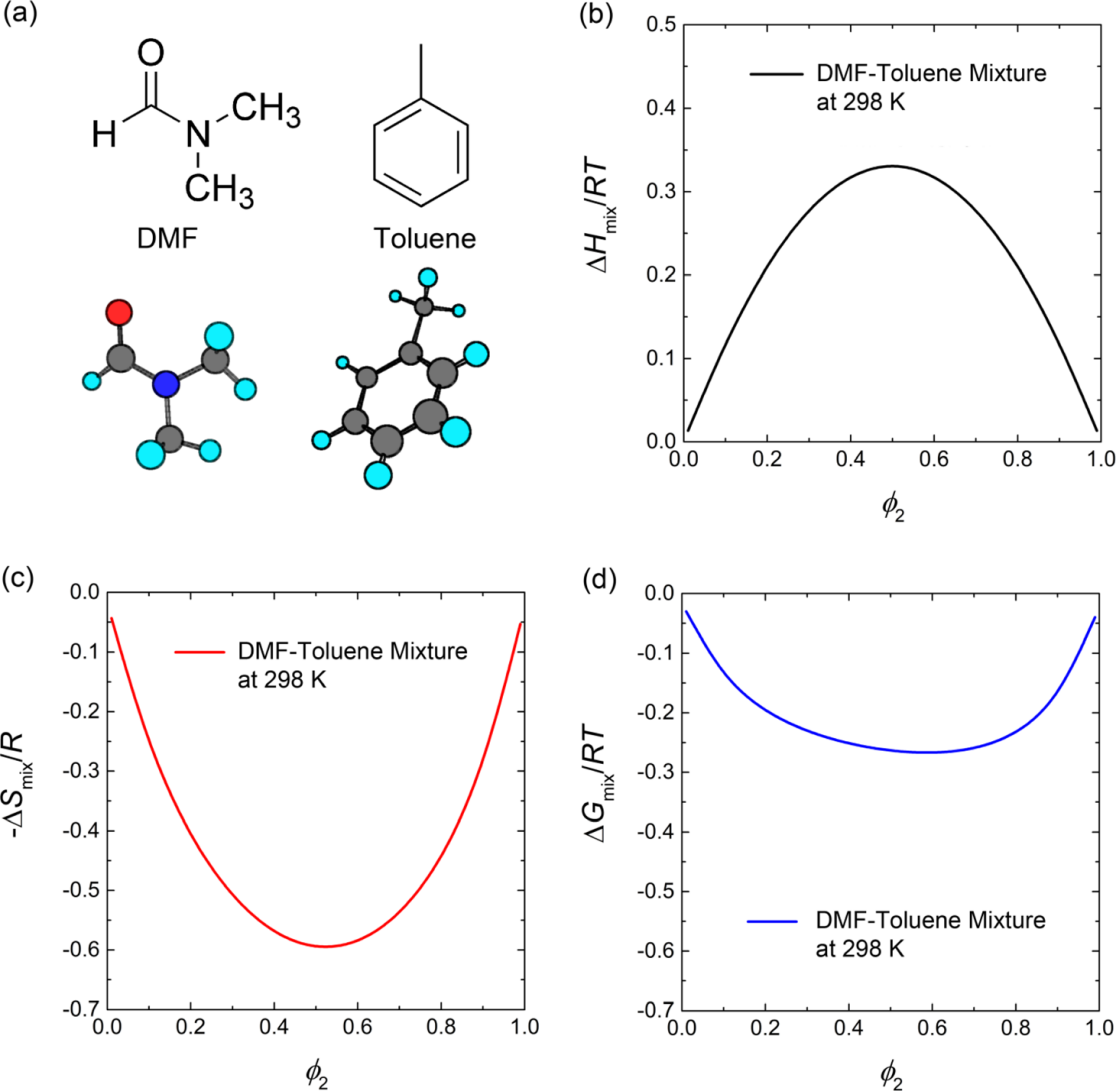
(a) Chemical structures of DMF and toluene. Prediction of Flory–Huggins theory for (b) enthalpy of mixing, (c) entropy of mixing, and (d) Gibbs free energy of mixing as a function of volume fraction of toluene, *ϕ*_2_ at 298 K.

To understand the miscibility between the polar solvent DFM and the nonpolar antisolvent toluene (Fig. 3a) in the SR-RT process, we employ the Flory–Huggins theory (reduced to the regular solution theory when two solvents are equal in molar volume) as follows,^38,47,48^3
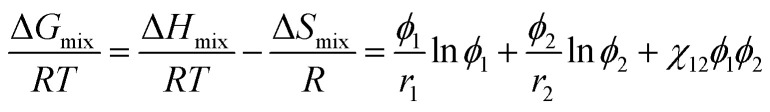
where Δ*G*_mix_ is Gibbs free energy of mixing, Δ*H*_mix_ is the enthalpy of mixing, Δ*S*_mix_ is the entropy of mixing and *χ*_12_ is the Flory–Huggins interaction parameter. *ϕ*_1_ and *ϕ*_2_ are the volume fractions of DMF and toluene, whereas *r*_1_(=1) and *r*_2_ are the relative molar volumes of DMF and toluene, respectively. Note that when *r*_2_ is 1, eqn (1) is reduced to the regular solution theory. However, in this work, the molar volumes of DMF and toluene are 77.4 cm^3^ mol^−1^ and 106.3 cm^3^ mol^−1^, respectively. Hence, we used eqn (3) with *r*_2_ = 1.37 = 106.3/77.4 (=toluene/DMF, molar volume ratio). Here, the interaction parameter (*χ*_12_) between DMF and toluene is defined as4
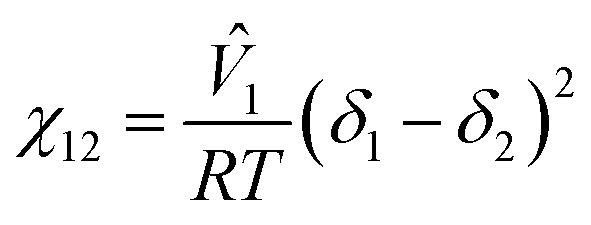
where *V̂*_1_ is the molar volume of DMF, whereas *δ*_1_ and *δ*_2_ are the solubility parameters of DMF and toluene, respectively. For example, *χ*_12_ is 1.34 when *V̂*_1_ is 77.4 cm^3^ mol^−1^, *R* = 1.987 cal K^−1^ mol^−1^, *T* = 298 K, *δ*_1_ = 12.1 cal^1/2^ cm^−3/2^ and *δ*_2_ = 8.9 cal^1/2^ cm^−3/2^. Accordingly, the enthalpy, entropy and Gibbs free energy of mixing were predicted for the binary DMF–toluene system (Fig. 3). First of all, the poor affinity between DMF and toluene opposes mixing by showing the positive enthalpy of mixing (Fig. 3b). However, because of the entropic gain (Fig. 3c), the Gibbs free energy of mixing decreases when DMF and toluene are mixed together as shown in Fig. 3d. Hence, when the toluene molecules were added to the perovskite precursor solution, they could replace DMF contacting the perovskite precursor molecules, resulting in the burst nucleation and growth of NCs *via* enhanced supersaturation. Note that through the water contact angle (*θ*_c_) data for the CsPbBr_3_ (bulk) and CsPbBr_3_ NC films, we can estimate their solubility parameters (see the ESI† section for details).^49,50^ For example, when *θ*_c_ = 10.57° for the CsPbBr_3_ film,^51^ the surface energy was calculated to be 71.594 mJ m^−2^, resulting in the solubility parameter *δ*_CsPbBr_3__ = 15.5 cal^1/2^cm^−3/2^ and 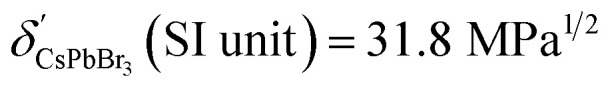. This estimation is based on the relation, 
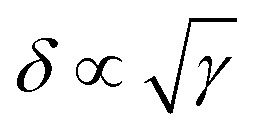
.^47,48,52^ In the case of CsPbBr_3_ NCs complexed with the surface ligands (OA and OAm), *θ*_*c*_ = 37.57°.^53^ Accordingly, the surface energy (*γ*) was estimated to be 61.603 mJ m^−2^, resulting in *δ*_CsPbBr_3_-NC_ = 14.4 cal^1/2^ cm^−3/2^ and 
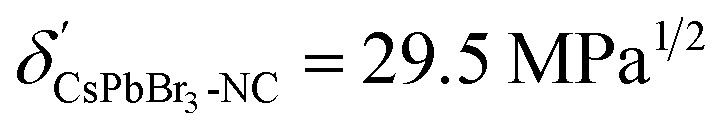
 for the CsPbBr_3_ NCs. Hence, the polarity of CsPbBr_3_ is partially reduced when complexed with OA and OAm. Note that OA and OAm have the solubility parameters of 7.9 cal^1/2^ cm^−3/2^ and 8.0 cal^1/2^ cm^−3/2^, respectively, exhibiting they are nonpolar, like antisolvent.^54^ However, the CsPbBr_3_-surface ligand complex is still polar, allowing the polar DMF to act as a good solvent. In addition, it is notable that DMF is a retrograde solvent,^55^ indicating that the degree of supersaturation will be enhanced with increasing temperature because the solubility of perovskite precursor in DMF will decrease with increasing temperature. Table 1 shows the summary of the solubility parameters related to this study.^56,57^

**Table 1:** Solubility parameter, molecular weight, density, and molar volume of the solvent, non-solvent, CsPbBr_3_ (bulk) and CsPbBr_3_ NC complexed with oleic acid and oleylamine. *δ*′ (SI unit) = *δ* × 2.0455

Molecule	*δ*′ (MPa^1/2^)	*δ* (cal^1/2^ cm^−3/2^)	MW (g mol^−1^)	*ρ* (g cm^−3^)	*V̂*_i_ (cm^3^ mol^−1^)
DMF	24.8	12.1	73.09	0.944	77.4
Toluene	18.2	8.9	92.14	0.867	106.3
Ethyl acetate	18.6	9.1	88.11	0.902	97.7
CsPbBr_3_	31.7	15.5	242	4.57	53.0
CsPbBr_3_ NC	29.5	14.4	242^a^	4.57^a^	53.0^a^

a Surface ligands are not considered.

**Fig. 4:**
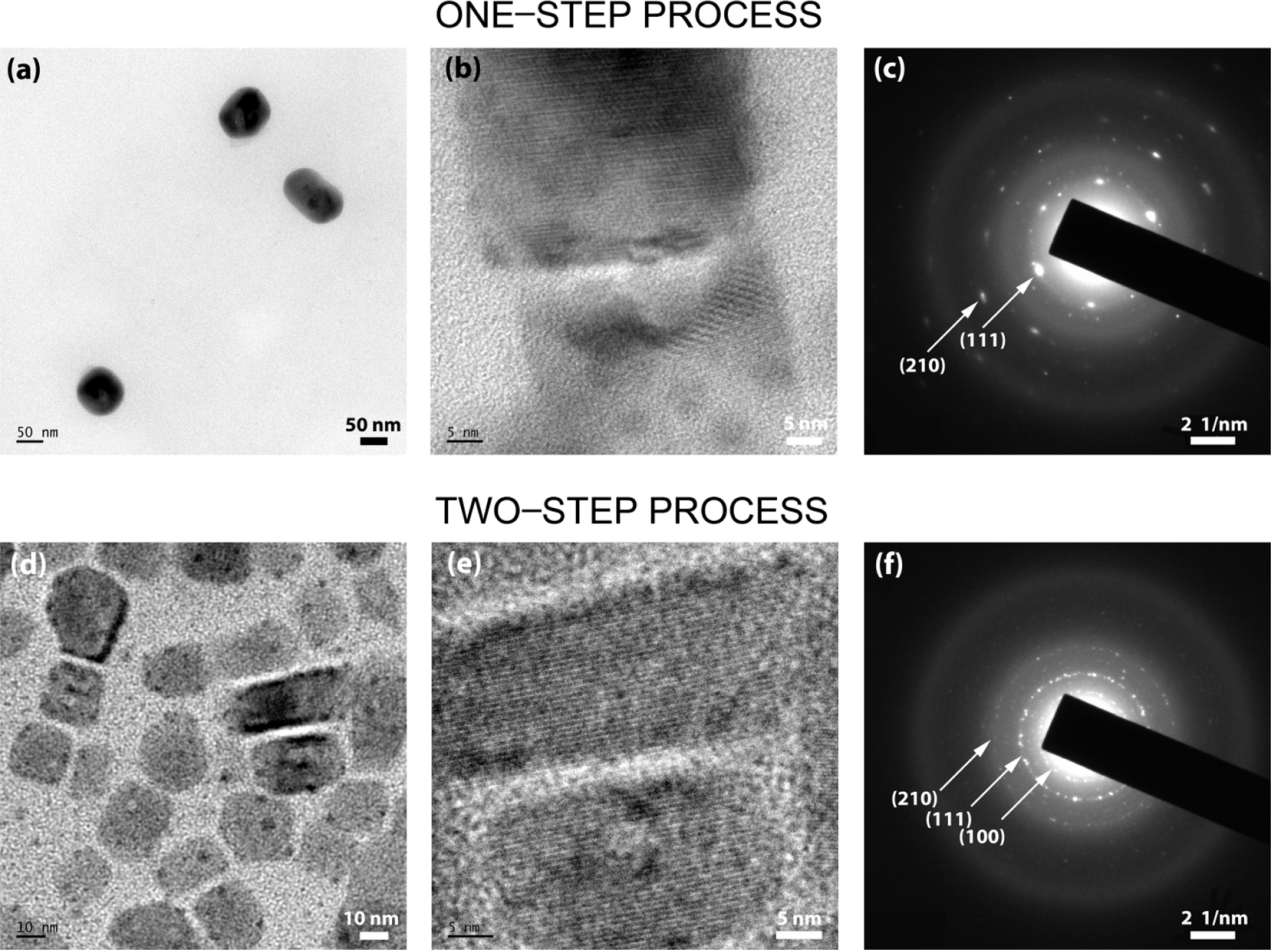
TEM images of CsPbBr_3_NCs synthesized through (a and b) one-step synthesis and (d and e) two-step synthesis. SAED images: (c) one-step synthesis and (f) two-step synthesis.

Fig. 4 shows the TEM images of CsPbBr_3_ NCs synthesized by one-step (Fig. 4a and b) and two-step (Fig. 4d and e) processes. In the one-step process, the average size of CsPbBr_3_ NCs is ∼60 nm, which is far from the excitonic confinement regime because its Bohr diameter is ∼7 nm. On the other hand, in the two-step process, the CsPbBr_3_ NCs exhibit a bimodal distribution (*i.e.*, two separate groups) with average NC sizes of ∼13.5 ± 2.5 nm (green emitter) and ∼3.5 ± 0.4 nm (blue emitter), respectively (Fig. S2†). The selected area electron diffraction (SAED) patterns (Fig. 4c and f) provide additional confirmation of the phases of the NCs. The one-step process displays the SAED pattern of a single crystal-like pattern, whereas the two-step process exhibits a polycrystal pattern because of two different NCs with versatile orientations.

Here, it is worth noting that in the one-step process, there is a high degree of supersaturation (unstable colloidal dispersion) allowing the perovskite precursor components (Cs^+^, Pb^2+^, Br^−^, and its complexes in the presence of OA and OAm) to form Cs[PbBr_3_] monomer aggregates resulting in the burst nucleation and fast growth of ∼60 nm sized NCs (Fig. 2b). Here, the entropy of mixing between DMF (∼1 mL) and toluene (10 mL) is the driving force (Fig. 3c) initiating the aggregate formation of Cs[PbBr_3_] monomers because of the repulsive interactions between the polar perovskite precursors and the nonpolar toluene. Interestingly, the phase inversion membrane in polymer science uses the same principle, *i.e.*, the exchange of antisolvent and solvent molecules for the membrane formation.^58^ On the other hand, in the case of the two-step process, the perovskite precursor molecules can contact the good solvent DMF molecules in the first stage (Fig. 2c) because it is approximately equal mixing between DMF (∼5 mL) and toluene (5 mL), resulting in a low degree of supersaturation, *i.e.*, relatively a limited formation of Cs[PbBr_3_] monomer aggregates allowing the formation of blue-emitting CsPbBr_3_ NCs. It is probably in a metastable state (or weak unstable state) because the larger green-emitting NCs were not allowed to be formed except for the smaller blue-emitting ones.

At this moment, if we employ the Hildebrand and Hansen solubility parameter,^37,56,57^ the supersaturation phenomena could be quantitatively analyzed during the one-step and two-step processes for the CsPbBr_3_ NC synthesis *via* the RT-SR processes. For DMF, *δ* = 12.1 cal^1/2^ cm^−3/2^, whereas for toluene, *δ* = 8.9 cal^1/2^ cm^−3/2^.^56,57^ The polar perovskite precursors (*δ* ∼ 14.4–15.5 cal^1/2^ cm^−3/2^) can aggregate easily in the nonpolar medium (toluene in the one-step process), whereas they can barely aggregate in the DMF–toluene (5 mL : 5 mL mixture) mixture with the average *δ*= (12.1 + 8.9)/2 ≈ 10.5 cal^1/2^ cm^−3/2^, *i.e.*, a (partially polar) marginal solvent. Hence, in the first stage of the ‘two-step process’, the nucleation and dissolution of CsPbBr_3_ could reach an unstable equilibrium (Fig. 2a), resulting in the blue-emitting CsPbBr_3_ NC formation with an average size of ∼3.5 nm. However, to collect these small blue-emitting NCs, some processing solvents, such as nonpolar ethyl acetate and/or toluene, should be used. Resultantly, the colloidal dispersion with the blue-emitting CsPbBr_3_ NCs may undergo additional burst nucleation and rapid growth of green-emitting CsPbBr_3_ NCs in spite of some depletion of monomers in the first stage, stipulating the purification step for separating the blue-emitters from the green ones.

**Fig. 5:**
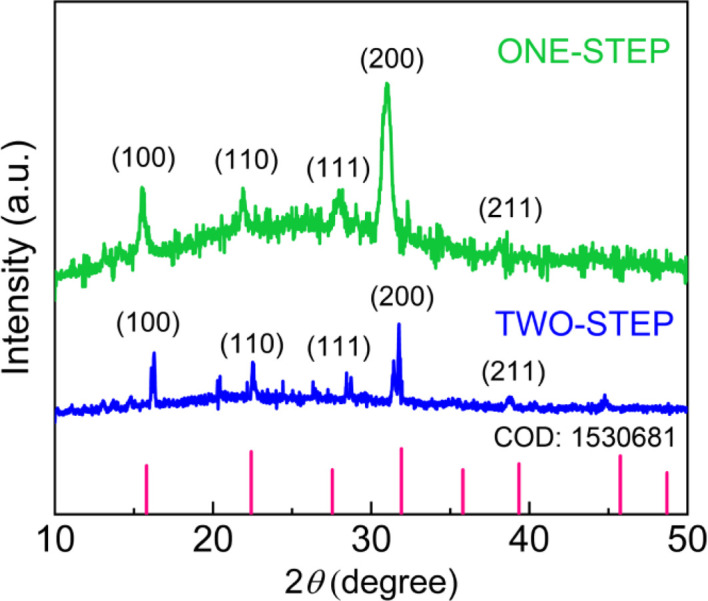
XRD patterns of CsPbBr_3_ NCs synthesized *via* the one-step and two-step SR method without purification.

XRD was employed to characterize the crystal structures of CsPbBr_3_ NCs. It has been reported that the tilt of corner-sharing {PbBr_6_}^4−^ octahedral building blocks causes CsPbBr_3_ to exist as polymorphs. The crystal phase symmetry of bulk CsPbBr_3_ increases with temperature, undergoing the phase transformation from orthorhombic to tetragonal (*P*4*mm*) at 88 °C and from tetragonal to cubic (*Pm*3*m*) at 130 °C.^47^ However, Fig. 5 clearly demonstrates that, due to the well-known high surface energy at the nanoscale, CsPbBr_3_ NCs have a cubic phase at RT by displaying the XRD peaks at 2*θ* = 16°, 22°, 28°, and 31°, which correspond to the (100), (110), (111), and (200) crystallographic planes, respectively.^59^ Importantly, in comparison to the one-step process, the two-step process exhibits slightly shifted XRD peaks to a higher degree, revealing a lattice contraction in the nanoscale particles with an edge size of ∼3.5–13.5 nm.^24^

**Fig. 6:**
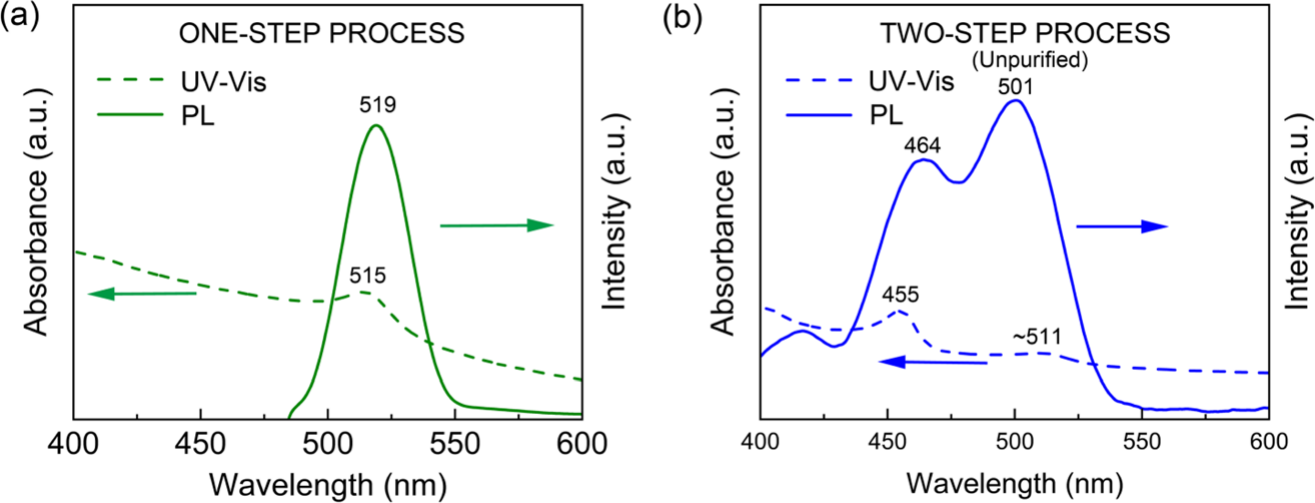
UV-vis and PL spectra of CsPbBr_3_ NCs: (a) one-step process and (b) two-step process without purification.

Fig. 6 shows the UV-Vis absorption and PL emission spectra for (a) one-step and (b) two-step processes before purification, respectively. Fig. 6a shows the absorption peak at 515 nm and the green-light emission peak at 519 nm with a full width at half maximum (FWHM) of ∼30 nm. Fig. 6b displays the two absorption peaks at 455 nm and ∼511 nm and the two PL emission peaks at 455 nm and ∼511 nm (the blue PL from ∼3.5 nm NCs and the green PL from ∼14 nm NCs), respectively (see Fig. S2† for NC's size distribution TEM data). The broad band spectrum of the unpurified two-step process samples is due to the wide range distribution of NCs from 2.5 nm to 25 nm, covering both the non-quantum confined (larger than 7 nm) and the quantum-confined (less than 7 nm) regions (see Fig. S2†).

**Fig. 7:**
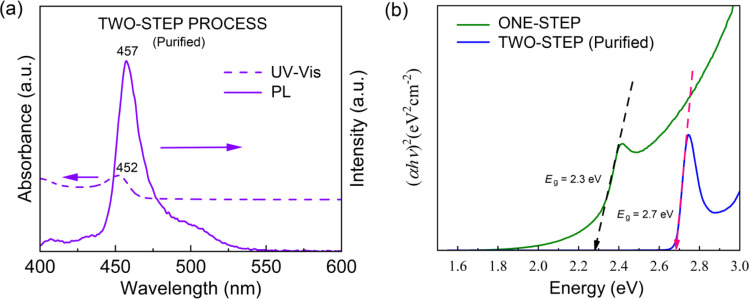
(a) UV-vis and PL spectra for the purified two-step synthesized CsPbBr_3_ NCs. (b) Tauc plot of one-step CsPbBr_3_ NCs and two-step CsPbBr_3_ NCs after purification.

Then, for collecting the blue-emitting CsPbBr_3_ NCs, we purified the two-step processed samples (Fig. 4d and e). Fig. 7a shows the UV-Vis absorption and PL emission spectra of the purified blue-emitting NC samples, displaying the absorption peak at 452 nm and the PL emission peak at 457 nm with FWHM of ∼23 nm. Here, this narrow FWHM data implies the relatively uniform distribution of CsPbBr_3_ NCs with a low trap density.^24,46^ Importantly, through the Tauc plot equation, (*αhν*)^*n*^ = *B*(*hν* − *E*_g_), we can quantify the optical bandgap (*E*_g_) of the NC samples (Fig. 7b). Here, *hν* is the photon energy, *α* is the absorption coefficient, *B* is a constant relative to the material, and *n* is either 2 for a direct transition or 0.5 for an indirect transition.^60^ As shown in Fig. 7b, the one-step and two-step processed samples exhibit an optical bandgap of 2.3 eV (green emitter) and 2.7 eV (blue one), respectively, demonstrating the process-nanostructure-optical property relationship.

**Fig. 8:**
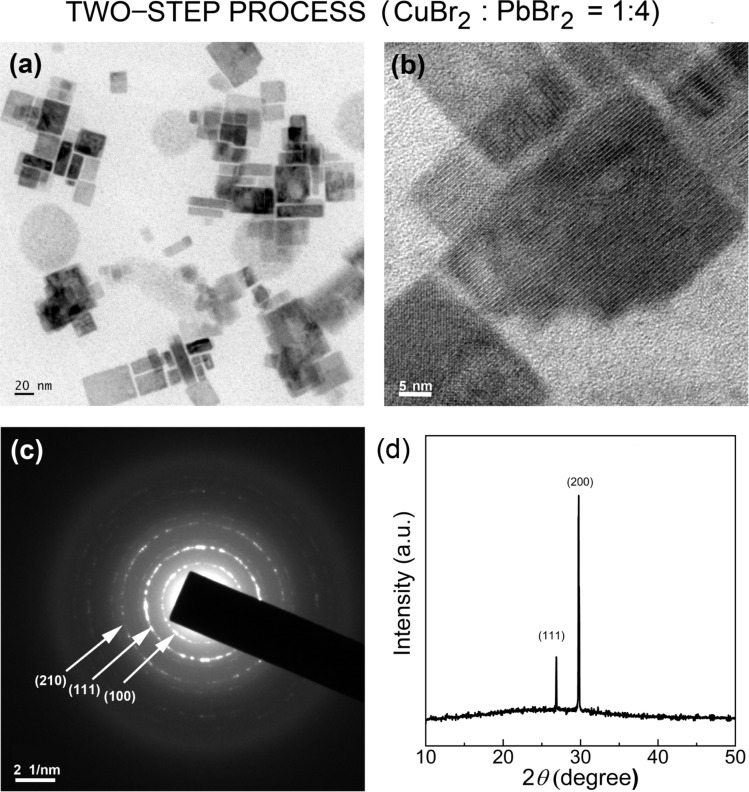
TEM images of CsPbBr_3_NCs synthesized through the two-step process with CuBr_2_ : PbBr_2_ = 1 : 4 mole ratio: (a) scale bar = 20 nm, (b) scale bar = 5 nm, and (c) SAED image. (d) XRD pattern of CsPbBr_3_NCs synthesized *via* a two-step process with CuBr_2_ : PbBr_2_ = 1 : 4 mole ratio.

We expanded the aforementioned two-step process by incorporating CuBr_2_ as an additive into the perovskite precursor solutions with CuBr_2_ : PbBr_2_ = 1 : 4 (molar ratio) for the first time *via* SR at RT. Here, by adding Cu^2+^ cations as well as Br^−^ anions with high Gutmann's donor number (*D*_N_ = 33.7, Lewis basicity),^61^ we may modify the interactions between perovskite precursor-solvent (DMF with *D*_N_ = 26.6; toluene with *D*_N_ = 0.1), affecting the crystallization process of LHPs.^50,61–63^Fig. 8a and b show the HR-TEM images of CsPbBr_3_ NCs processed with the CuBr_2_ additive, displaying the clear cubic NCs, indicating that the additive may stabilize the nanoscale cube with a sharp vertex in spite of its increased surface energy (compared to the spherical structure). Here, we observed again a bimodal distribution, composed of average ∼3.8 ± 0.7 nm and ∼21.4 ± 9.5 nm sized NCs (Fig. S3†). Note that this NC size is slightly larger than that of NCs without the additive in Fig. 4 and S2.† Furthermore, Fig. 8c shows the corresponding SAED image, indicating that the cubic CsPbBr_3_ NCs are oriented to the [110] direction on top of the TEM copper grid. On the other hand, the XRD pattern of the cubic CsPbBr_3_ NCs (Fig. 8d) displays two strong peaks at 26.8° and 29.9°, corresponding to (111) and (200) crystallographic planes, indicating that the cubic NCs are oriented more to the [200] direction on top of the glass slide. Hence, compared to the XRD in Fig. 5, the XRD pattern in Fig. 8d is much more simplified, indicating the enhanced orientational order when processed with the CuBr_2_ additive.

**Fig. 9:**
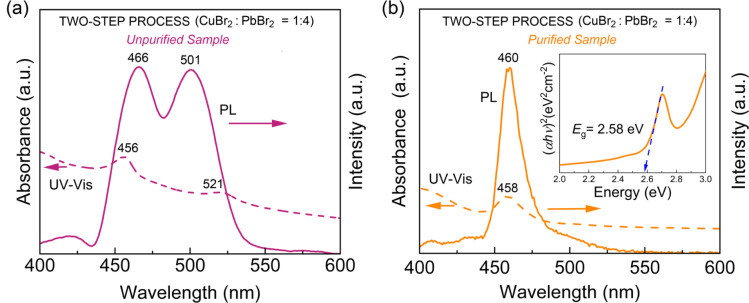
UV-vis and PL spectra of CsPbBr_3_ NCs synthesized through two-step synthesis with doping (CuBr_2_ : PbBr_2_ = 1 : 4 mole ratio): (a) unpurified sample and (b) purified sample. Inset: Tauc plot of the purified sample.

Fig. 9 shows the UV-Vis absorption and PL emission spectra for (a) the unpurified and (b) purified CsPbBr_3_ NCs with CuBr_2_. Compared to the unpurified sample in Fig. 6b (without CuBr_2_), the unpurified sample in Fig. 9a (with CuBr_2_) shows a slight red-shift (UV-Vis absorption peaks from ‘455 nm and 511 nm’ to ‘566 nm and 521 nm’ whereas PL emission peaks from ‘464 nm and 501 nm’ to ‘466 nm and 501 nm’) (compare Fig. 6b and 9a). The same trend was also observed in the purified samples by displaying the minor red shift (UV-Vis absorption from 452 nm to 458 nm whereas PL emission from 457 nm to 460 nm). This red shift could be rationally understood by observing the partial increase of the CsPbBr_3_ NC size in the presence of the CuBr_2_ additive, as shown in Fig. S1 and S6.† Furthermore, it is interesting to observe the reduced PL FWHM of ∼18.5 nm (Fig. 9b), which is significantly smaller than ∼23 nm in Fig. 7 (without CuBr_2_), indicating that the size-focusing effect is available when processed with the CuBr_2_ additive. However, because of the partial increase of NC size, the optical bandgap is accordingly reduced from 2.7 eV (Fig. 7b) to ∼2.6 eV (see the inset in Fig. 9b), displaying the quantum size effect.

**Fig. 10:**
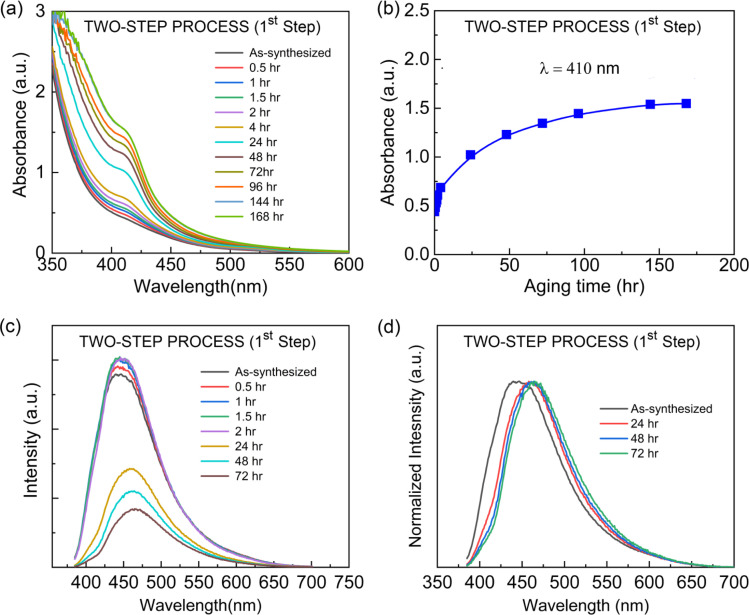
Aging effect on the optical properties of CsPbBr_3_ NCs (after the first step in the ‘two-step process’) at room temperature. (a) UV-vis absorption spectra as a function of aging time at room temperature. (b) The absorption at *λ* = 410 nm. (c) PL emission spectra as a function of aging time at room temperature. (d) Normalized PL intensity as a function of aging time at room temperature.

The two-step process produces a bimodal distribution, as demonstrated through the TEM data in Fig. 4d, S2, 8a, and S3.† Hence, it would be valuable to examine the stability and crystallization of the original solution samples after the first stage ‘separately’ in the two-step process. For this purpose, after the initial mixing of ‘the precursor solution in 5 mL DMF’ and ‘5 mL toluene’, the solution was stored for aging effect at RT – this is a low degree of supersaturation state. Fig. 10 shows the UV-Vis absorption and PL emission spectra as a function of aging time at RT. Fig. 10a displays clearly that the absorption increases with time. However, based on the peak's location, we determined that there is only the blue-emitting CsPbBr_3_ NCs without the Oswald ripening effect related to the green emitter growth (the quenching mechanism is simply all monomer consumption at RT). Furthermore, when the UV-Vis absorption data was plotted at the wavelength (*λ*) of 410 nm with aging time, a saturation behavior was observed after ∼50–100 hours (Fig. 10b). Subsequently, we examined the PL emission behavior as a function of aging time. As shown in Fig. 10b, the PL increases initially but decreases after 24 hours, indicating NCs might be deactivated, *i.e.*, quenching the PL emission. Furthermore, the PL red-shift was observed, indicating a partial growth of blue-emitting CsPbBr_3_ NCs, but still, it is a blue emitter, indicating that there might be a balance between formation and dissolution of the blue-emitting NCs in this low degree of supersaturated state. However, recall that for collecting these NCs, additional nonpolar antisolvent should be required as a processing solvent, resulting in another nucleation and growth of green emitters from the new monomer aggregates generated by the enhanced degree of supersaturation (Fig. 2c). Moreover, in the two-step process (1st step), the UV-Vis and PL spectra display that the CsPbBr_3_ NCs grow with a wide range of size distribution (Fig. 10). However, still, the majority of the NCs are in a quantum-confined regime. However, when the antisolvent toluene is additionally added for the second time, it may cause some of the NCs to aggregate and grow, resulting in non-quantum confined NCs. Hence, two competing peaks were observed in Fig. 6b.

**Fig. 11:**
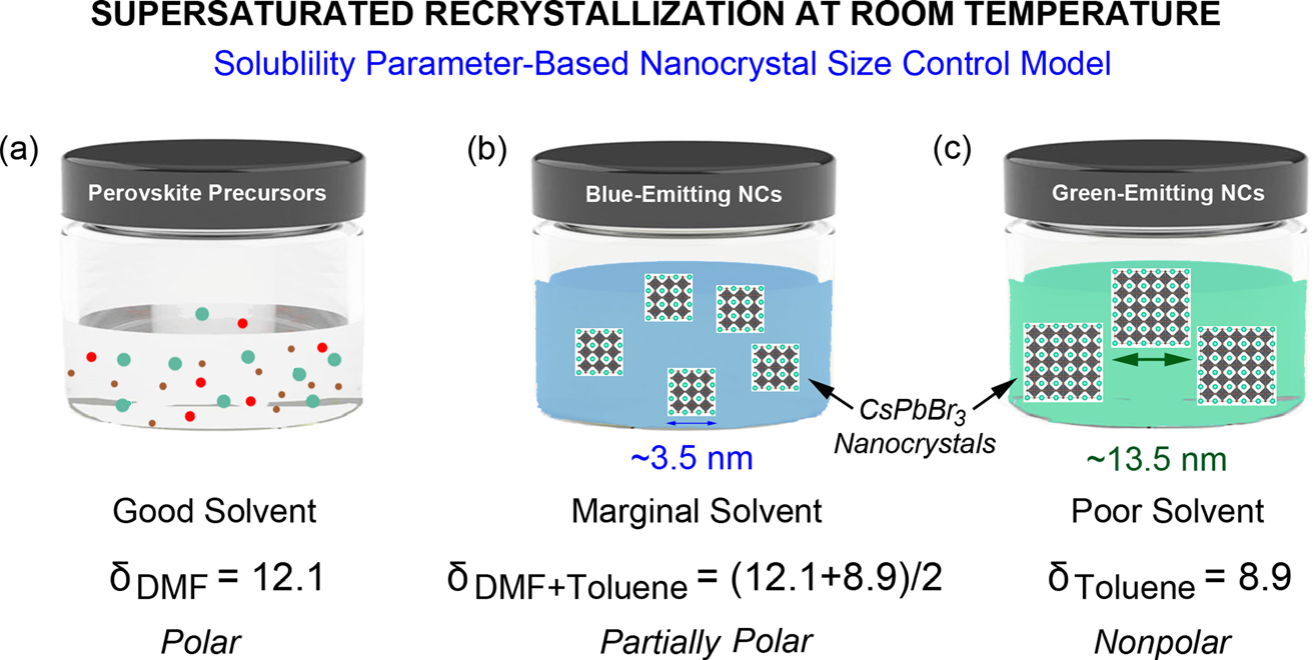
Supersaturated recrystallization at room temperature: solubility parameter-based nanocrystal size control model: (a) good solvent = polar, (b) marginal solvent = partially polar, and (c) poor solvent or antisolvent = nonpolar.

Fig. 11 is the summary of this work suggesting ‘the solubility parameter-based nanocrystal size control model’ for explaining the SR process at RT. When the perovskite precursors are in a good solvent like DMF, there is no nucleation and growth of NCs because they can stay as a well-dispersed colloidal system. However, by adding an antisolvent (toluene) into the perovskite precursor solution in DMF with a ∼50 : 50 volume ratio, the liquid medium becomes a marginal solvent with partial polarity, resulting in the burst nucleation and rapid growth of blue-emitting CsPbBr_3_ NCs. Here, the DMF–toluene 50 : 50 mixture provides a significant solubility for colloidal perovskite precursors (Fig. 2c), and no sufficient monomer aggregates remain for further growing to green emitters at this low supersaturation level. Finally, if the liquid medium is dominantly nonpolar (toluene ≫ DMF), the green-emitting NCs can be easily grown in the presence of blue-emitting NCs. Hence, the solubility parameters (or solubility) of the solvent and solvent–antisolvent mixture can serve as a key factor for the nanoscale size regulation in the SR process at RT, affecting (1) the nucleation and growth of NCs and (2) the balance between formation and dissolution of Cs[PbBr_3_] monomers and NCs in line with the LaMer diagram in Fig. 2 and 11.

## 5. Conclusions

The nucleation and growth of CsPbBr_3_ NCs were studied in view of the classical LaMer model when the supersaturated recrystallization was carried out at room temperature. For this purpose, we compared one-step and two-step processes to elucidate the formation mechanism of NCs. Resultantly, when the nonpolar toluene (antisolvent) was much larger in volume than the polar DMF (solvent), the ∼60 nm sized green-emitting NCs were synthesized *via* the one-step process under a high degree of supersaturation. On the other hand, when the antisolvent volume is comparable to that of the solvent, the ∼3.5 nm-sized blue-emitting NCs were synthesized *via* a two-step process using a low degree of supersaturation. In this study, by employing the Hildebrand and Hansen solubility parameter concept, we quantitatively explained the solvent quality (good, marginal, and poor). Hence, we named this approach ‘the solubility parameter-based nanocrystal size control model’ because supersaturation is a function of ‘solubility and polarity’ (expressed as solubility parameter) and ‘antisolvent–solvent volume ratio’ (considered as average solubility parameter). Furthermore, based on the Flory–Huggins model, we predicted that the antisolvent–solvent mixing is driven by the entropy of mixing, allowing the crystallization of the perovskite precursor to aggregate *via* SR operating at RT. Finally, in the presence of the CuBr_2_ additive in the two-step process, we observed a partial red-shift in both absorption and emission spectra from the quantum size effect (a slight increase of nanocube). In addition, the PL emission spectra showed a reduced PL FWHM of ∼18.5 nm (reduced size scattering and enhanced size focusing) from the ∼3.8 nm NCs. Finally, we believe that our finding, *i.e.*, the importance of the solubility parameter as an NC size control factor for the SR method at RT, will contribute to further advancing the perovskite nanocrystal technology and beyond. Future work may include the formation mechanism of NCs *via* SR-RT as a function of the average solubility parameter from the versatile antisolvent–solvent mixtures.

## Author contributions

Writing – original draft preparation, D. A. I.; experiments and formal analysis, D. A. I., M. A., D. M., J. K. P., L. T., A. T., S. T. and J. Y. K.; and writing – review and editing, J. Y. K.

## Conflicts of interest

The authors declare no competing financial interest.

## Supplementary Material

NA-006-D4NA00423J-s001
