# Supplementary material for: Cesium lead bromide perovskite nanocrystals synthesized via supersaturated recrystallization at room temperature: comparison of one-step and two-step processes
Source: Nanoscale Adv. 2024 Jun 18;6(16):4137–48. doi: 10.1039/d4na00423j (PMC11302073; doi:10.1039/d4na00423j)
Supplement: NA-006-D4NA00423J-s001 [file NA-006-D4NA00423J-s001.pdf]

Electronic Supplementary Material (ESI) for Nanoscale Advances.
This journal is © The Royal Society of Chemistry 2024

Supplementary Information

Cesium Lead Bromide Perovskite Nanocrystals Synthesized by Supersaturated Recrystallization at Room Temperature: Comparison of One-Step and Two-Step Processes

Dula Adugna Idosa, [a,b] Mulualem Abebe,[a] Dhakshnamoorthy Mani,[a] Jibin Keloth Paduvilan,[c] Lishin Thottathi,[d] Aparna Thankappan,[e] Sabu Thomas[f] and Jung Yong Kim *,[g,h]

[a] Faculty of Materials Science and Engineering, Jimma Institute of Technology, Jimma University, Jimma P.O. Box 378, Ethiopia

[b] Department of Physics, College of Natural and Computational Science, Mizan-Tepi University, Mizan P.O. Box 260, Ethiopia

[c] School of Chemical Sciences, Mahatma Gandhi University, Kottayam 686560, India

[d] Department of Physics and Mathematics, Università Cattolica del Sacro Cuore, Via della Garzetta, 48, 25133 Brescia, BS, Italy

[e] Department of Physics, Baselius College, Kottayam 686001, India

[f] School of Energy Materials, Mahatma Gandhi University, Kottayam 686560, India

[g] Department of Materials Science and Engineering, Adama Science and Technology University, Adama P.O. Box 1888, Ethiopia

[h] Center of Advanced Materials Science and Engineering, Adama Science and Technology University, Adama P.O. Box 1888, Ethiopia

* Correspondence: jungyong.kim@astu.edu.et
ORCID ID: https://orcid.org/0000-0002-7736-6858

Electronic Structures of $CsPbBr_3$ Perovskite:

Density functional theory (DFT)-based first-principle calculations were carried out to determine electronic structures of $CsPbBr_3$ perovskites. The cubic phase of $CsPbBr_3$ belonging to the space group Pm3m with (No.221) was taken into account. Figure S1a shows the band structure of $CsPbBr_3$'s unit cell with cubic structure. Here, both the valence band maximum (VBM) and conduction band minimum (CBM) were located at the k-point **R**, signifying a direct bandgap semiconductor. The bandgap (E_g) was estimated to be 1.44 eV, which is smaller than ~2.3 eV of the experimental result. However, it is consistent with the literature reports based on the *generalized gradient approximation* (GGA) with *Perdew-Berke-Ernzerhof for solids* (PBEsol), i.e., (PBEsol_GGA) method (the typical underestimation, i.e., smaller value, of bandgap).[S1.S2] Figure S1b demonstrates the projected density of states (PDOS) in which the VBM is dominated by $4p^5$ whereas the CBM by $6p^2$ orbitals.

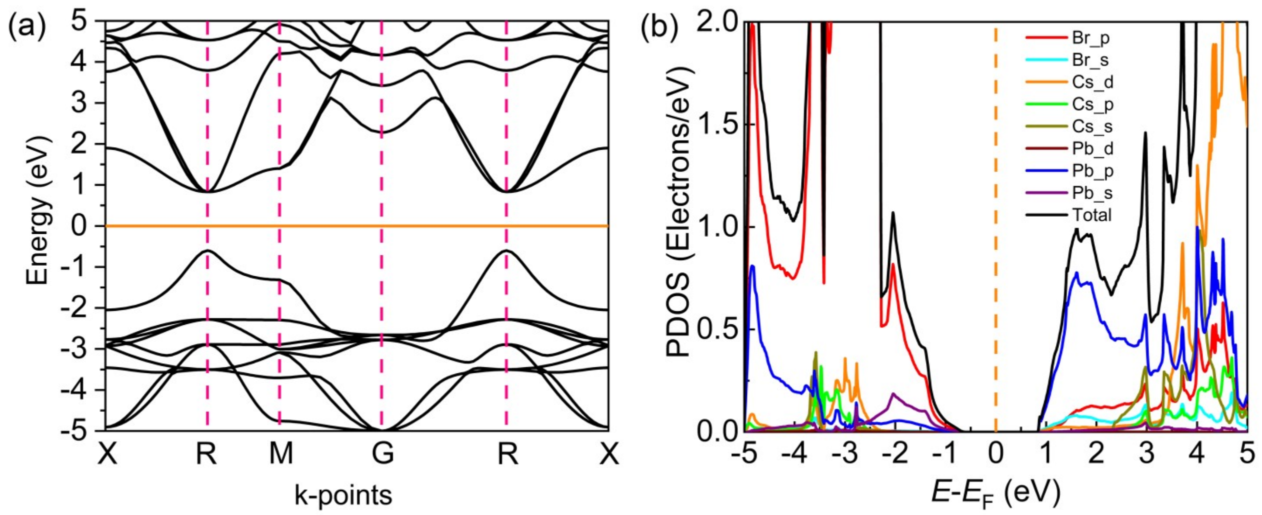

Figure S1. (a) Electronic band structure of $CsPbBr_3$. (d) Projected density of states (PDOS) of $CsPbBr_3$.

Conversion of Water Contact Angle to Surface Energy to Solubility Parameter:

According to Li and Neumann, the water contact angle (θ_c) has a following relationship,

$$\cos\theta_c = -1 + 2\sqrt{\gamma_{sv}/\gamma_{lv}} \cdot \exp\left[-\bar{\beta}\left(\gamma_{lv} - \gamma_{sv}\right)^2\right] \qquad \text{(S1)}$$

where, γ_{lv}, γ_{sv} and γ_{sl} are surface energies for liquid-vapor, solid-vapor, and solid-liquid, respectively, whereas the constant $\bar{\beta}$ is 0.000115 m^4 mJ^{-2}, and γ_{lv} = 72.8 mJ m^{-2} for water.[S3] Hence, when θ_c = 10.57° for $CsPbBr_3$,[S4] γ_{sv} = 71.59391 mJ m^{-2} is calculated by solving Equation (S1) via the Newton-Raphson method. Then, through the relationship $\delta_2\left[\text{cal}^{1/2}\text{cm}^{-3/2}\right] = 1.829058\sqrt{\gamma_{sv}}$,[S5] δ_{CsPbBr_3} is estimated to be 15.5 $cal^{1/2}cm^{-3/2}$. In the same vein, when θ_c = 37.57° (processed via toluene) and 23.69° (processed via dodecane) for $CsPbBr_3$ NCs,[S6] γ_{sv} = 61.60304 mJ m^{-2} and γ_{sv} = 61.59607 mJ m^{-2}, resulting in δ_{CsPbBr_3-NC} =14.356≈14.36 $cal^{1/2}cm^{-3/2}$ and δ_{CsPbBr_3-NC} =14.355 ≈14.36 $cal^{1/2}cm^{-3/2}$, respectively. Interestingly, the quasi 2D hybrid lead bromide perovskite ($BA_2FA_{n-1}Pb_n\ Br_{3n+1}$, n → 1; BA and FA stand for n-butyl ammonium and formamidinium, respectively) was reported to have the solubility parameters of from 15.38 $cal^{1/2}cm^{-3/2}$ to 15. 60 $cal^{1/2}cm^{-3/2}$ depending on the processing conditions, verifying the aforementioned data[S4,S6] are reasonable values.[S7]

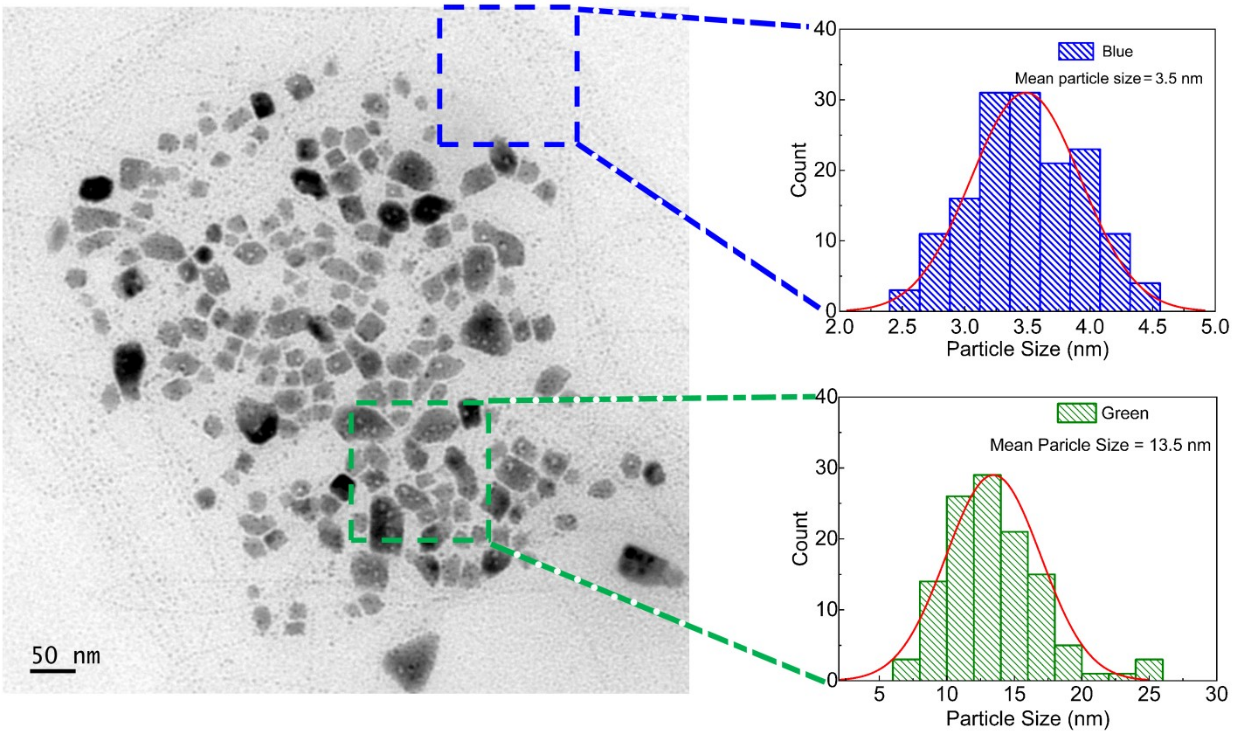

Figure S2. TEM image of unpurified two-step synthesized $CsPbBr_3$ NCs showing particles distribution. Blue-emitting NC size: ~ 3.5 ± 0.4 nm; Green-emitting NC size: ~13.5 ± 3.5 nm.

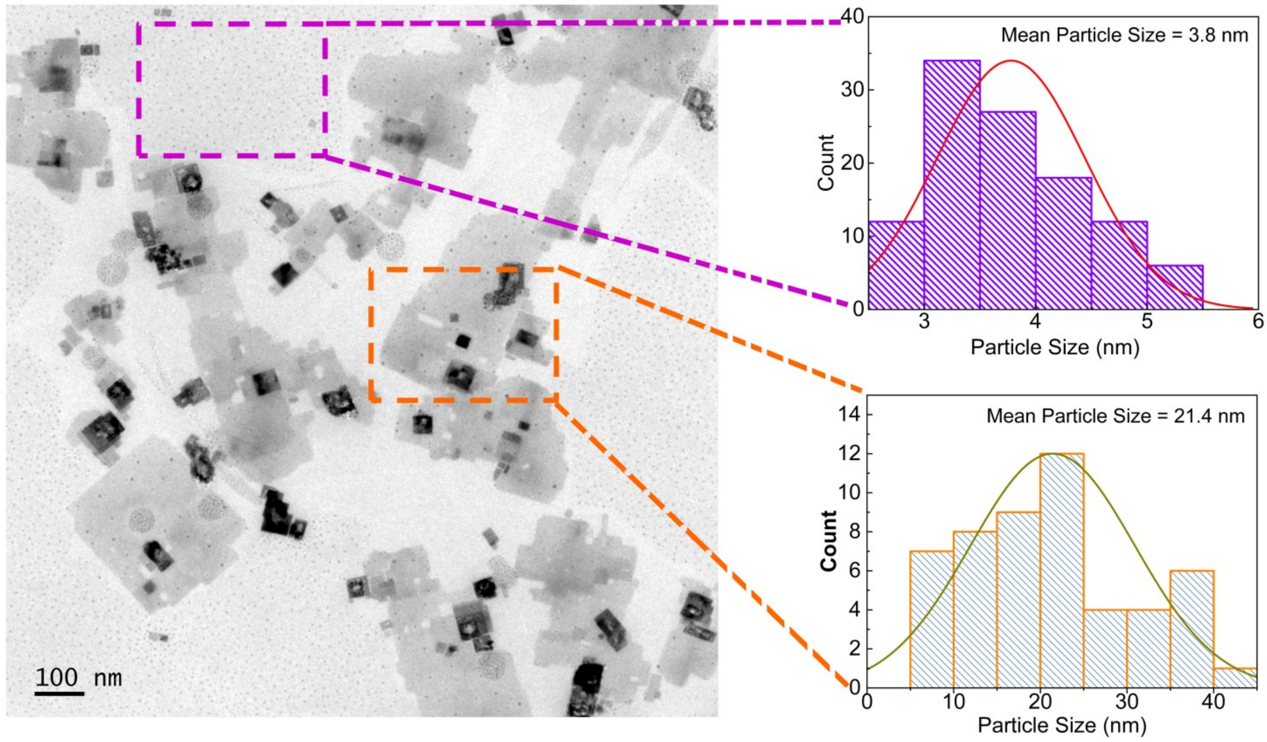

Figure S3. TEM images of $CsPbBr_3$ NCs with $CuBr_2$ (unpurified) synthesized by two-step process with $CuBr_2$:$PbBr_2$ (= 1:4 molar ratio) displaying a bimodal distribution of NCs. Blue-emitting NC size: ~ 3.8 ± 0.7 nm; Green-emitting NC size: ~21.4 ± 9.5 nm.

References

S1. D. Yan, T. Shi, Z. Zang, T. Zhou, Z. Liu, Z. Zhang, J. Du, Y. Leng and X. Tang, *Small*, 2019, **15**, 1901173.

S2. Y. Zhang, G. Li, C. She, S. Liu, F. Yue, C. Jing, Y. Cheng and J. Chu, *Nano Res.*, 2021, **14**, 2770–2775.

S3. D. Li, A.W. Neumann, *J. Colloid Interface Sci.*, 1990, **137**, 304-307.

S4. X. Zeng, W. Li, C. Yan, J. Cao, X. Fu and W. Yang, *J. Mater. Chem. C*, 2021, **9**, 15967-15976.

S5. S. Nilsson, A. Bernasik, A. Budkowski, E. Moons, *Macromolecules*, 2007, **40**, 8291–8301.

S6. A. Gao, J. Yan, Z. Wang, P. Liu, D. Wu, X, Tang, F. Fang, S.Ding, X. Li, J. Sun, M. Cao, L. Wang, L. Li, K, Wang and X. W. Sun, *Nanoscale*, 2020,**12**, 2569-2577.

S7. J.Y. Kim, Y. Yoo, J. Kim, H. J. Park, W. Cho, S. Lee, Y.-E. Sung and S. Bae, *ACS Appl. Opt. Mater.*, 2024, **2**, 108-117.
